# Automatic Concrete Damage Recognition Using Multi-Level Attention Convolutional Neural Network

**DOI:** 10.3390/ma13235549

**Published:** 2020-12-05

**Authors:** Hyun Kyu Shin, Yong Han Ahn, Sang Hyo Lee, Ha Young Kim

**Affiliations:** 1 Architectural Engineering, Hanyang University, ERICA, Ansan 15588, Korea; hyunkew@hanyang.ac.kr (H.K.S.); yhahn@hanyang.ac.kr (Y.H.A.); 2 Division of Smart Convergence Engineering, Hanyang University, ERICA, Ansan 15588, Korea; 3 Graduate School of Information, Yonsei University, Seoul 03722, Korea

**Keywords:** concrete defects, damage recognition, convolutional neural network, deep learning, attention network

## Abstract

There has been an increase in the deterioration of buildings and infrastructure in dense urban regions, and several defects in the structures are being exposed. To ensure the effective diagnosis of building conditions, vision-based automatic damage recognition techniques have been developed. However, conventional image processing techniques have some limitations in real-world situations owing to their manual feature extraction approach. To overcome these limitations, a convolutional neural network-based image recognition technique was adopted in this study, and a convolution-based concrete multi-damage recognition neural network (CMDnet) was developed. The image datasets consisted of 1981 types of concrete surface damages, including surface cracks, rebar exposure and delamination, as well as intact. Furthermore, it was experimentally demonstrated that the proposed model could accurately classify the damage types. The results obtained in this study reveal that the proposed model can recognize the different damage types from digital images of the surfaces of concrete structures. The trained CMDnet demonstrated a damage-detection accuracy of 98.9%. Moreover, the proposed model could be applied in automatic damage detection networks to achieve superior performance with regard to concrete surface damage detection and recognition, as well as accelerating efficient damage identification during the diagnosis of deteriorating structures used in civil engineering applications.

## 1. Introduction

Old buildings and infrastructure in dense urban regions have been consistently exposed to aggressive environmental conditions. These deteriorated components degrade the structural performance, simultaneously revealing several types of defects, such as cracks, delamination, and rebar exposure, on the surfaces of concrete structures. The superficial damage of a concrete structure is an intuitive indicator of its condition and serviceability. Thus, it is important to obtain accurate information about the superficial damage of concrete structures. However, the current visual inspection approach for investigating the conditions of buildings, which is conducted manually by inspectors, is extremely costly and labor-intensive. Moreover, it is time-consuming to detect damage and determine the type of damage that has occurred [1,2,3,4,5,6].

To overcome these limitations, several researchers have attempted to replace the existing damage detection methods with computer vision-based approaches. The main advantage of computer vision-based damage recognition is that it enables the identification and automatic classification of superficial structural defects [3,7].

In this context, the image processing technique (IPT) has been widely explored in combination with various computing technologies for the inspection and monitoring of structural safety [8]. To identify the characteristics of surface damage on concrete components, as well as on other materials, previous researchers have proposed damage detection methods based on several techniques, such as the Gabor filter histogram of gradient (HOG) [5], the support vector machine [9], beamlet transform, thresholding, and edge detection methods [2,3,10]. For instance, Yeum and Dyke [5] proposed an image feature extractor using the HOG technique to identify crack damage near bolts on steel structures. Ying and Salari [11] presented a beamlet transform-based technique to extract linear crack features from pavement images. Furthermore, German et al. [2] introduced a local entropy-based threshold to extract a spall damage map. Koch and Brilakis [3] proposed automated pothole detection in asphalt pavement images using a histogram shape-based threshold. In addition, Zalama et al. [10] presented a road-surface-crack classifier that employs Gabor filters to detect longitudinal and transverse cracks. Several other researchers have attempted to construct models for pavement crack analysis based on image threshold approaches [1,4,11,12,13,14].

The results of these previous studies have demonstrated that each method could detect and identify specific damages in structures, such as asphalt pavement, bridges, and buildings. However, an IPT-based classifier requires the selection of representative thresholds that uniquely represent the object without being affected by variations in aspects such as position, scale, lighting, and background [5]. Furthermore, determining the representative threshold for damage recognition is challenging because it depends on the type of damage and on the geometric patterns represented on the surface of the structure [15]. Since common superficial concrete images are exposed in various forms in real concrete structures, the performance of image processing techniques is influenced not only by various types of noise, such as dust and spots, but also by brightness [15]. Although various studies have been conducted to determine a threshold for image classification, image processing techniques still have limitations in real-world situations owing to their manual feature extraction approach [1,7,16].

To overcome these challenges, various deep learning models have recently been proposed to represent the features of concrete structural damage automatically based on digital images [7,13,17,18]. The convolutional neural network (CNN)-based approach is an advanced image analysis technique applied to various domains, which achieves remarkable performance in terms of addressing complex problems in image recognition. Since a convolutional filter is only slightly influenced by noise caused by environmental variations, CNN-based models can identify and classify images accurately. 

Therefore, several CNN-based image classification and recognition techniques have been developed for various purposes; however, they have mostly been focused on a specific type of damage, particularly crack damage. Furthermore, the deteriorated structures in actual environments not only exhibit crack damage but also experience delamination, leakage, and rebar exposure on their surfaces. [15]. Since damage to concrete structures occurs in various forms, research on multi-damage classification is necessary for practical application. 

This paper proposes a method of analyzing images containing various types of damages and describes the empirical experiments conducted for concrete surface damage identification using CNNs. The proposed model was designed to improve performance by recognizing several forms of damage. The main contribution of this work is that it provides a multi-damage recognition network to classify the types of damages automatically. In addition, various architectures are discussed to explore the structures that are the most likely to experience concrete surface damage. Finally, an improved design with good performance is proposed, combined with an attention network module and hybrid pooling layers. The improved design is described in detail in the subsequent sections.

## 2. Related Work

In the past, several convolution-based image analysis techniques were developed to improve the performance of models by solving vision problems. In existing studies, deeper networks have been proposed to handle large-scale image datasets. For instance, the AlexNet proposed by Krizhevsky [19] has eight layers, including five convolutional layers and three fully connected layers with a 224 × 224 × 3 input image. In contrast, the VGG16 model consists of 16 layers [20]. The architecture of the VGG model is similar to that of the AlexNet; however, the former has a deeper layer, and the main similarity is that small and uniform convolution filters are used in all convolution layers. The use of small filters allows the VGG model to have a large number of layers. In addition, it has been demonstrated that a deeper CNN improves its performance in image classification tasks [20]. Furthermore, various strategies, such as the inception block [21] and shortcut connection [22] approaches, have been adopted to achieve robust deep-layer architectures. 

In the building and civil engineering domain, several researchers have applied CNN models to address the limitations of the visual inspection process in terms of maintenance and management. For instance, Cha et al. [7] proposed a crack detection approach comprising convolution and pooling layers using concrete surface image data. Nhat-Duc [13] proposed a CNN-based automatic pavement-crack-detection model to identify whether the images have crack damage or not. Furthermore, Yang et al. [18] proposed a fully convolutional network to recognize a crack and to calculate the length and width of the crack damage by dividing the images into pixels. Wang et al. [17] proposed a CNN-based damage classification model to replace the conventional human-based visual inspection of old concrete and brick buildings. They also effectively combined the CNN model with the sliding window technique.

Lin et al. [16] developed a CNN technique optimized for image analysis. It is resistant to noise interference and automatically extracts damage features from low-resolution images of structure surfaces. Yang et al. [18] proposed a fully convolutional network (FCN) for computer-image-based analysis. It is capable of investigating and classifying cracks appearing on the surfaces of structures into pixels. Moreover, the FCN could calculate the dimensions of the cracks detected from digital images, although the corresponding accuracy achieved lay in the −13.27–24.01% range. This implies that models that are more robust should be developed to realize superior performance.

As already mentioned, several models have been proposed to develop an advanced concrete surface damage recognition model based on computer vision data by applying both image processing and deep learning techniques. In addition, since CNN models are optimized for image analysis, they have been mainly used to clarify various features on the surfaces of structures, and several experiments have been conducted to fit target datasets [17,18]. However, previous studies have focused exclusively on crack detection. Accordingly, they have limited applicability with regard to simultaneously analyzing the different damage types that appear on the surfaces of structures.

To conduct multi-damage image analysis, an optimized CNN capable of processing various damage types needs to be explored because diverse and complicated damage types occur in real-world situations. Therefore, a model capable of multi-damage analysis was developed in this study, and its performance was experimentally investigated by applying various models. The proposed CMDnet model adopts the existing approach to recognizing surface damage from images using a CNN-based deep learning algorithm. However, compared to the previous model, CMDnet can simultaneously classify up to five damage types. The performance of the proposed model can be improved by combining the attention network that can be learnt by emphasizing the features automatically extracted while passing through the multi-level in the convolution neural network. Moreover, to prevent overfitting, the proposed model recognizes different damage types by replacing the max-pooling layer with a hybrid pooling layer. The proposed model architecture is described in detail in the subsequent sections.

## 3. Methodology

This section describes the proposed method, which employs attention network branches in the multi-level convolution neural network. Additionally, the refinement of the pooling layer using the hybrid pooling module is described herein. The experiments performed in this study reveal that the attention network and hybrid pooling play a significant role in improving the prediction performance of CMDnet.

### 3.1. Proposed Model Architecture

**Figure 1:**
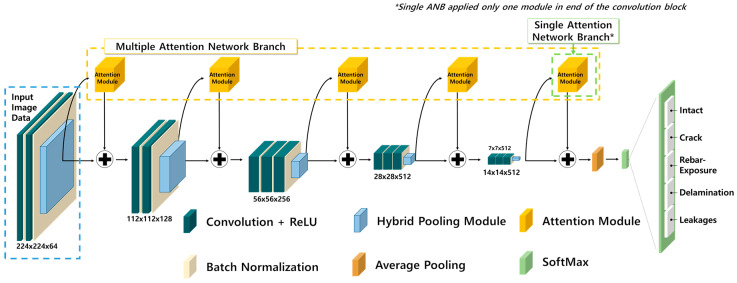
Convolution-based multi-damage recognition neural network for concrete structures.

In this study, a CNN was applied to analyze a multi-damage dataset in order to classify the input images into five categories (i.e., non-damage, cracks, delamination, leakage, and rebar exposure) and to construct a convolution-based concrete multi-damage recognition neural network (CMDnet). Figure 1 depicts a schematic of the proposed model. This network is designed based on VGG16 [20], and it combines the auxiliary layers with a hybrid pooling layer and attention network modules. The main objectives of the proposed model are to extract sensitive feature maps and to provide a robust means of handling concrete surface damage. 

### 3.2. Hybrid Pooling 

**Figure 2:**
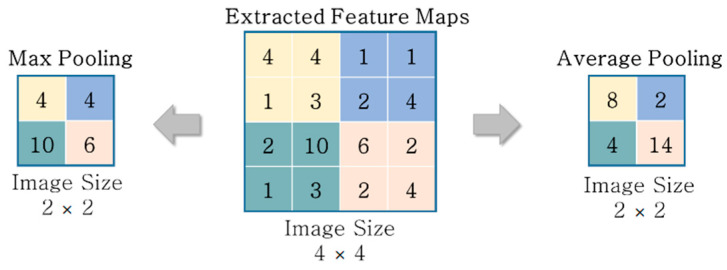
Examples of maximum and average pooling.

The main role of the pooling layer is to reduce the position resolution when the result of the convolution operation is used as input data, selecting the active neuron of each region [23,24]. The objective of the pooling layer is to achieve robustness to illumination changes and position variations with invariance to feature transformations [25]. In practice, most incipient CNNs have employed popular pooling methods, such as average pooling and maximum pooling [26]. Average pooling considers all the elements in the pooling regions to prevent variance increases while retaining the background information [26,27], whereas maximum pooling only captures the foreground texture information of the strongest activation as a representative feature of a region of an image, as shown in Figure 2 [24].

**Figure 3:**
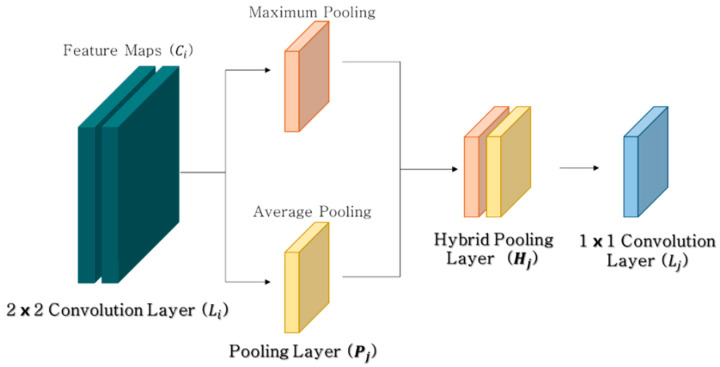
Hybrid pooling modules combining maximum and average pooling.

However, Boureau et al. [28] noted that there are some drawbacks, as maximum and average pooling may lose information representing the background and foreground, respectively [26]. Thus, a concatenated pooling method combining the characteristics of maximum and average pooling was utilized in this study to prevent information loss near the representative features because of the spatial characteristics of concrete surface damage. Therefore, hybrid pooling can also improve the robustness of the concrete damage recognition method during the training process. Figure 3 shows a schematic of the hybrid pooling module.

Hybrid Pool(*H_j_*) = Concatenate (Max Pool_j_(*C_i_*), Avg Pool_j_(*C_i_*)) (1)

where $C_{i}$ is a feature map extracted from the convolutional function in the previous layer ($L_{i}$), and Concatenate (a, b) are two input values attached to each other. The hybrid pooling layer can prevent the loss of small but significant information by harnessing the advantages of maximum and average pooling. Concrete damage recognition tasks are sensitive to local transformations of the input images; therefore, maximum pooling stacked with average pooling can retain all the information in each region of a complex image. 

### 3.3. Attention Network

**Figure 4:**
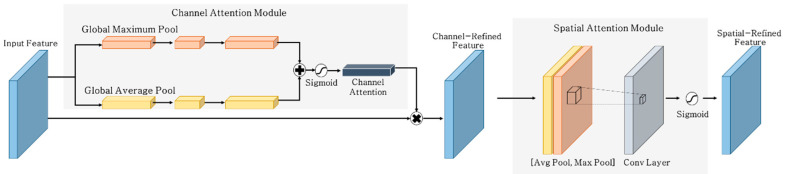
Attention network architecture.

The attention network (see Figure 4) mechanism plays a role in enhancing the operation of feature extractors, improving the image recognition accuracy by focusing on essential features in the process of learning the image features [29,30,31]. Wang et al. [29] proposed a residual attention network, which is a CNN combined with an attention module. Whenever the training image data passes through the block-stacked attention modules, the critical features that are represented at each level are strengthened. Park et al. [30] further proposed a bottleneck attention module (BAM) and refined the process of extracting features by separating channel and spatial information and extracting the main features. The BAM consists of a channel attention branch, spatial attention branch, and a combination of two branches. The feature extractor contains multiple fully connected layers for identifying inter-channel relationships and multiple convolution layers for focusing spatial location information. Another separated attention mechanism is the convolutional block attention module (CBAM) proposed by Woo et al. [31]. The CBAM improves utilization compared to be BAM by separating the channel and spatial attention modules and can be inserted in residual form between convolution blocks. The channel attention module adopts both maximum and average pooling to achieve realize channel-based attention. The spatial attention module of the CBAM also applies maximum and average pooling. This module is similar to the afore-described hybrid pooling approach, but the spatial pooling in the CBAM computes the mean according to the channel axis. Thus, the feature map size is retained, even passing through the spatial attention module. Therefore, the CBAMs proposed in [31] were applied to the deep CNN (DCNN) model in this study, which enabled the architecture to represent high response features in concrete damage recognition when applied to the image data. 

## 4. Implementation

### 4.1. Establishing Concrete Surface Damage Dataset

In this study, we constructed a concrete structure damage dataset by collecting data corresponding to four representative superficial defects (i.e., cracks, delamination, leakage, and rebar exposure) for multi-damage recognition. The 4032 × 3024 px resolution images were obtained by examining the defects in deteriorated concrete structures using a digital camera. There exist no specific standards regarding camera specifications to be considered. In this study, all images were captured using a generic digital camera and smartphone with a resolution of 4032 × 3024 px. All images depicting structural damage were resized to 224 × 224 px during preprocessing. The number of images depicting concrete damage equaled 1981 images.

**Figure 5:**
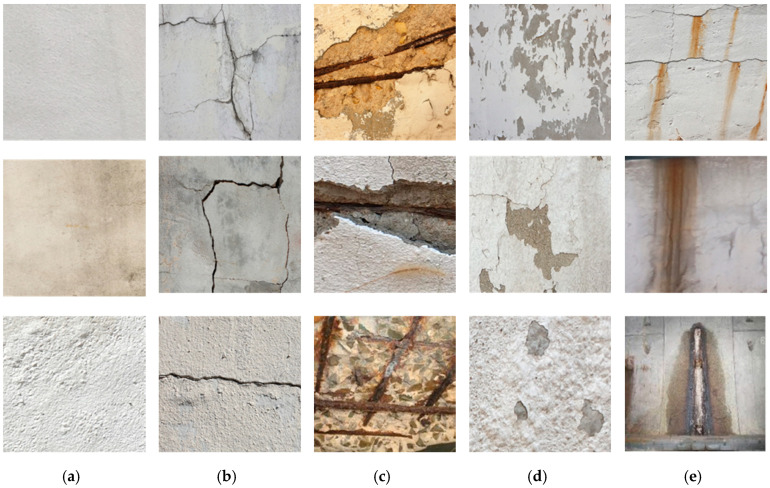
Examples of concrete damage. (a) Intact; (**b**) Crack; (**c**) Rebar exposure; (**d**) Delamination; (**e**) Leakage.

Figure 5 shows a representative image of each damage type, which can be clearly distinguished by the naked eye. 

The size of the raw image is 4032 × 3024 px, but it was resized to 224 × 224 px to fit the proposed model. To prepare the training dataset, we classified the data into the following categories: crack (530), delamination (563), rebar exposure (268), and leakage (208), as well as non-damage (412) images. However, approximately 2000 images are not sufficient to train a DCNN and achieve excellent performance in damage classification. To improve the performance and achieve more robust models, larger and different datasets are required [19]. In a low-data regime and conditions under which the collection of extensive datasets is limited, this approach has limitations in terms of improving the performance because of underdetermined parameters [32]. To overcome these drawbacks, data augmentation strategies are essential for fine-tuning deep networks [33]. Many researchers have proposed data augmentation strategies for efficient training in networks. Krizhevsky et al. [19] proposed image translations and horizontal and vertical reflections to prevent overfitting. The use of these image transformations could reduce the error rate. Another approach proposed by [34] involves developing a statistical model of the transformations to implement augmentation schemes using training data; they demonstrated that this approach has some advantages compared with manual specification. Therefore, data augmentation strategies were adopted in this study to prevent overfitting during the training process. This approach improves training accuracy without additional training data through image transformations, such as horizontal/vertical reflection, random brightness, rotation, zoom, and cropping within a defined range [19].

### 4.2. Experimental Settings

The experiments in this study were conducted using the Keras API platform with a customized CNN-based model. As afore-described, the representative CNN models (i.e., AlexNet, InceptionV3, ResNet50, VGG16, and MobileNetV2) were adopted as baselines for classifying concrete damage recognition. The experiments were conducted using the Keras platform on a workstation with a GPU (GeForce GTX 1080Ti) and CPU (Intel Core i9-7980XE CPU, 2.60 GHz × 18). To identify the optimal architectures on the concrete damage dataset, we conducted a preliminary examination using AlexNet, VGG16, ResNet50, InceptionV3, and MobileNetV2. 

During the experiments performed in this study, the dataset was divided into training and test data in a 9:1 ratio. Thus, the training and test datasets contained 1785 and 196 images, respectively. In the training process, the training and validation datasets contained 1430 and 355 images, respectively, during the preprocessing stage. The remaining 196 images (10%) were used for testing the proposed model.

A common problem in DCNN training is that the hyperparameters are highly sensitive; thus, the network was trained using the Adam optimizer [35] with a learning rate of 0.0001 for a total of 5000 epochs. For the best performance of the experimental models, we monitored the validation loss per epoch and updated the weight variables when the loss decreased during the training process. Subsequently, performance evaluations were performed using a test set.

## 5. Results and Discussion

### 5.1. Performance Evaluation Metrics 

To measure the performance of the model, we used accuracy as the model performance metric across all predictions [7]. In this study, accuracy was defined as the ratio of the number of correct answers to the entire test dataset:

(2) $$\mathrm{Accuracy}\;=\;\frac{\mathrm{True Positive}\;+\;\mathrm{True Negative}}{\mathrm{Total Samples}}$$

where True Positive is the number of matches of actual positive and predicted positive values, and True Negative is the number of matches of actual negative and predicted negative values. In other words, it evaluates how many correctly predicted classes make up the entire actual class. 

However, accuracy is not the preferred performance measure for classifiers, particularly when dealing with very imbalanced test data. A more suitable means of assessing the performance of a classifier involves evaluating the precision, recall, and F1-score, whose respective equations are as follows [7,13].

(3) $$\mathrm{Precision}\;=\;\frac{\mathrm{True Positive}}{\mathrm{True Positive}\;+\;\mathrm{False Positive}}$$

(4) $$\mathrm{Recall}\;=\;\frac{\mathrm{True Positive}}{\mathrm{True Positive}\;+\;\mathrm{False Negative}}$$

(5) $$F1\text{-}\mathrm{Score}\;=\;2\cdot \frac{\mathrm{Precision}\cdot \mathrm{Recall}}{\mathrm{Precision}\;+\;\mathrm{Recall}}$$

Therefore, in this study, four metrics were used to evaluate model performance: accuracy, precision, recall, and F1-score. 

### 5.2. Experimental Results

To compare the performance of the proposed CMDnet with other CNN models, 196 testing images, which were different from the training and validation datasets, were prepared. After training for 5000 epochs (445,000 iterations), the trained models were evaluated using the test dataset. The precisions, recalls, and F1-scores of the experimental models for the testing images are summarized in Table 1. The experimental results show that the proposed model achieved 98.98% accuracy, which represents the best performance among the experimental models in terms of recognizing damage types. 

**Figure 6:**
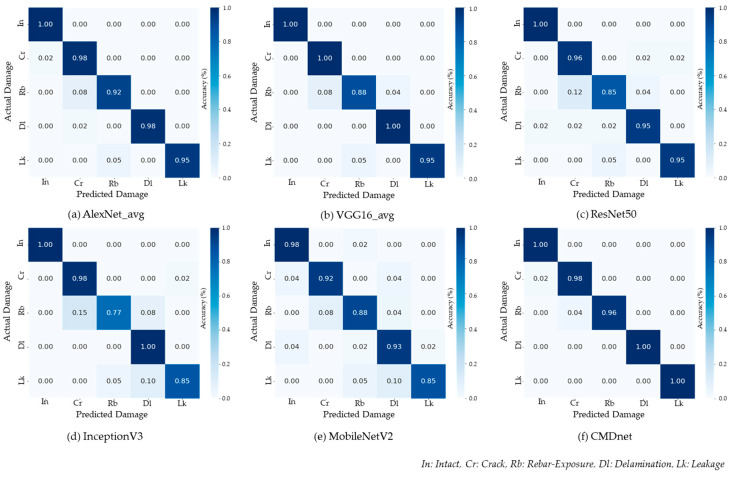
Normalized confusion matrices.

To analyze the recognition accuracy of each model for each damage type, we constructed the confusion matrices and receiver operating characteristic (ROC) curves. In the experiment, the predicted output was the type of damage with the highest probability among the five categories, and the accuracy was calculated based on the number of correct predictions. Figure 6 shows the confusion matrices describing the prediction accuracy according to the classification type of each model. The proposed model achieved a concrete damage recognition accuracy of at least 96%.

**Figure 7:**
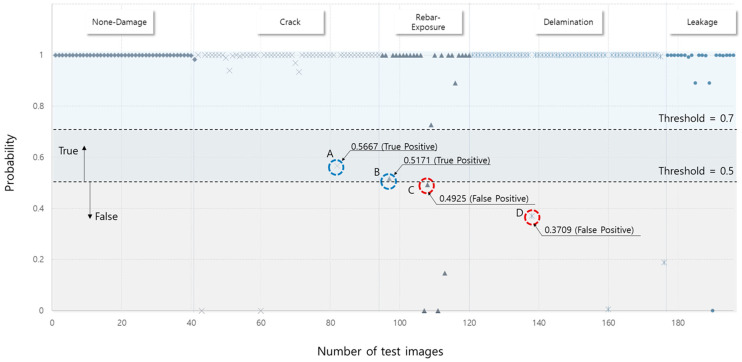
Prediction probability distribution with cut point located at 0.7; results A and B were found to be false positive but were determined to be true positive in model-performance evaluation.

**Figure 8:**
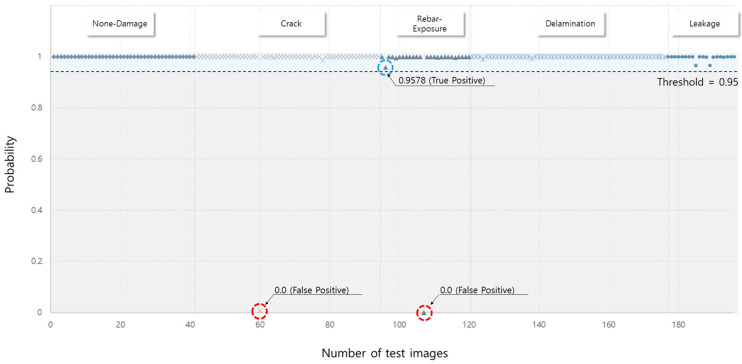
Prediction probability distribution of proposed model.

However, it is difficult to identify the internal performance because the prediction result was determined based on the highest probability among the five categories, and was identified as a true positive; even if the prediction probability is less than 70%, it could be considered as a true positive. For example, although the prediction probability is approximately 50% for the prediction results in Figure 7, the model accuracy is unaffected because the results yielded a value higher than the other prediction values. On the other hand, as depicted in Figure 8, the convolution-based CMDnet can accurately classify multiple damages with at least 95% probability.

**Figure 9:**
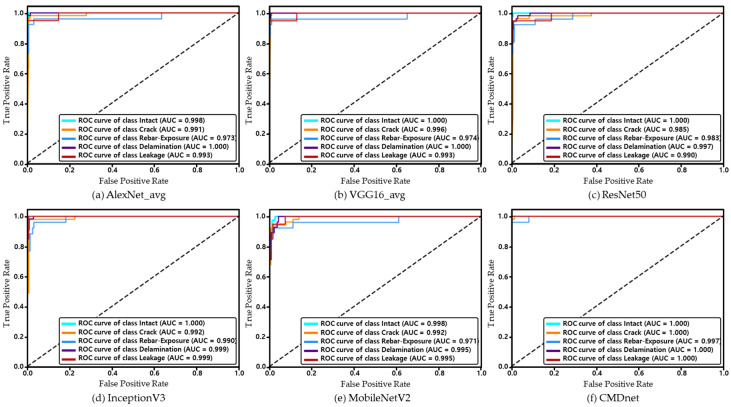
ROC curves for different models.

**Figure 10:**
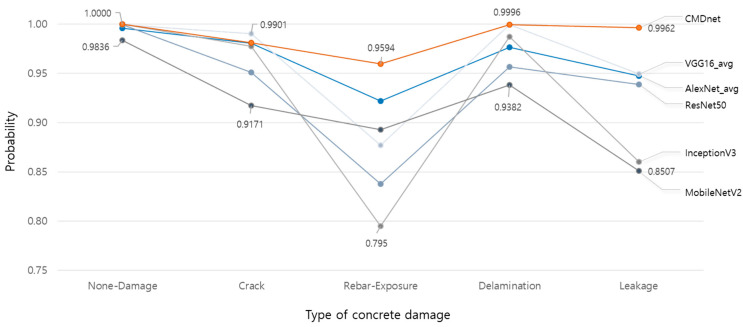
Average prediction probability of different models according to concrete damage type.

Therefore, the ROC curve was used to evaluate the performance of the model in more detail. Figure 9 shows the ROC curves of the models tested in this study. The area under the curve (AUC) is a metric for evaluating model performance based on the predicted probability by calculating the false-positive ratio and percentage of correct answers. The higher the AUC, the better the performance. Compared to other models depicted in Figure 10, CMDnet demonstrated a reliable performance, thereby achieving test results wherein the correct damage type was identified in nearly all images. Thus, compared to previous models, the proposed method can accurately predict the different damage types appearing on the surfaces of concrete structures.

## 6. Conclusions and Scope for Future Work

The various types of damages that occur on concrete surfaces represent the conditions of building structures. Thus, structural condition diagnosis is essential to assess structural durability. In the conventional identification and diagnosis method, inspectors investigate superficial damage. However, to automate the evaluation of the conditions of buildings and infrastructure, the recognition of various types of damage information must be performed. 

To achieve high-performance automatic concrete damage recognition, CMDnet was developed in this study. CMDnet provides the automatic multiple damage classification of concrete surface images obtained from deteriorated buildings and infrastructure using a CNN feature extractor. Unlike previous models, the proposed model adopts an attention network and hybrid pooling in the convolution block with batch normalization, and various damage types can be identified and distinguished accurately. Furthermore, the proposed model can automatically classify the different shapes and types of damage occurring on concrete surfaces in a practical environment. Accordingly, the proposed model attains a higher prediction accuracy compared to previously proposed techniques. 

**Figure 11:**
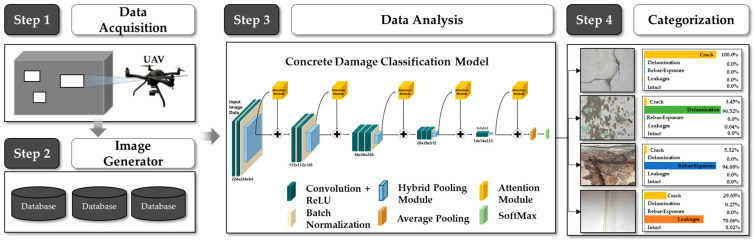
Conceptual diagram of concrete damage inspection process.

Concrete surface multi-damage recognition using CMDnet achieved a 95.7% minimum probability of correct prediction, with a 98.9% accuracy. To increase the application of automatic recognition models in building and civil engineering, the convolution-based customized CMDnet is proposed to accelerate efficient damage type identification in the post-processing of the diagnosis of deteriorating structures. Moreover, the proposed model could be applied in automatic damage detection networks to achieve superior performance in terms of concrete surface damage detection. Furthermore, the proposed method can be used as a part of the method for inspecting the exterior of concrete structures using a drone, as depicted in Figure 11.

A major limitation of the proposed model encountered when performing image classification is that it cannot visually confirm the location of the information being analyzed during damage detection from a given image. In addition, the methods capable of predicting numerical information—length, width, and area—necessary for assessing the condition of a building were excluded from this study.

In future endeavors, the authors intend to focus on a visualization model capable of verifying the proposed model’s ability to perceive structural damage accurately. Further, the upgraded model would be able to capture numerical information to facilitate the quantitative evaluation of the damage for automatic condition assessment.

## Figures and Tables

**Table 1:** Overall accuracies, precisions, recalls, and F1-scores of Convolutional Neural Network(CNN)-based experimental models.

Models	Accuracy (%)	Precision (%)	Recall (%)	F1-score (%)
AlexNet_avg *	0.97449	0.97449	0.974490	0.97449
VGG16_avg *	0.97959	0.97943	0.974489	0.97692
ResNet50	0.94879	0.95408	0.948979	0.95149
InceptionV3	0.94879	0.94898	0.948979	0.94898
MobileNetV2	0.92347	0.92824	0.923469	0.92582
Proposed model—CMDnet	0.98980	0.98980	0.989780	0.98978

* AlexNet_avg and VGG16_avg transposed the end of the three layers from the fully connected layer to the average pooling layer.
